# MYC/MAX-Activated LINC00958 Promotes Lung Adenocarcinoma by Oncogenic Transcriptional Reprogramming Through HOXA1 Activation

**DOI:** 10.3389/fonc.2022.807507

**Published:** 2022-02-09

**Authors:** Tao Zhang, Fei Su, Yong-bin Lu, Xiao-ling Ling, Huan-yu Dai, Tian-ning Yang, Bin Zhang, Da Zhao, Xiao-ming Hou

**Affiliations:** ^1^ Department of Oncology, The First Hospital of Lanzhou University, Lanzhou, China; ^2^ The Second Clinical Medical College of Lanzhou University, Lanzhou, China; ^3^ Department of Lung Cancer, Tianjin Medical University Cancer Institute and Hospital, National Clinical Research Center for Cancer, Key Laboratory of Cancer Prevention and Therapy, Tianjin’s Clinical Research Center for Cancer, Tianjin Lung Cancer Center, Tianjin, China

**Keywords:** lung adenocarcinoma, LINC00958, MYC, MAX, transcriptional reprogramming, HOXA1

## Abstract

**Background:**

Lung adenocarcinoma (LUAD) is the most common histological subtype of lung cancer. The role of the long non-coding RNA (lncRNA) LINC00958, which regulates the malignant behavior of multiple tumors, in LUAD has not been elucidated.

**Methods:**

Tissue microarray, FISH, and qRT-PCR were used to detect the expression of LINC00958. Plasmid and viral infections were used to manipulate gene expression. The role of LINC00958 in LUAD was studied by cell proliferation analysis, cell apoptosis analysis, cell migration and invasion analysis, and subcutaneous inoculation of animal models. At the same time, RNA-Seq, RNA pull-down, ChIRP, ChIP, and luciferase reporter gene assays were performed to clarify the mechanism.

**Results:**

The expression of LINC00958 in LUAD tissues was significantly upregulated when compared with that in adjacent tissues and could independently predict poor survival of patients with LUAD. LINC00958 knockdown significantly inhibited the growth and metastasis of lung cancer cells *in vitro* and *in vivo*. LINC00958 localized to the nucleus, regulated oncogenes and metabolism-related and immune response-related genes, and interacted with histones. The targets of LINC00958 were *TRPV3*, *STAP2*, and *EDN2* promoters with motifs of HOXA1, NANOG, FOSL2, JUN, and ATF4. Moreover, HOXA1 overexpression mitigated the LINC00958 knockdown-induced oncogenic phenotype. MYC/MAX motif, which was detected at the cis-element of LINC00958, trans-activated the LINC00958 promoter.

**Conclusions:**

MYC/MAX-trans-activated LINC00958 promotes the malignant behavior of LUAD by recruiting HOXA1 and inducing oncogenic reprogramming.

## Introduction

Lung cancer is associated with the highest rates of incidence and mortality among all cancers in both males and females worldwide (1). The most common histological subtype of lung cancer is lung adenocarcinoma (LUAD) (2). Small-molecule tyrosine kinase inhibitors and immunotherapy have increased the survival rates of patients with LUAD (3). However, the overall survival and resolution rates are low in patients with advanced LUAD. Therefore, there is a need to elucidate the molecular mechanisms underlying LUAD pathogenesis and identify novel and efficient therapeutic targets to improve the prognosis of patients with LUAD.

Long non-coding RNAs (lncRNAs), which have a length of more than 200 nucleotides, do not encode proteins. However, lncRNAs regulate protein translation through different mechanisms, including epigenetic, transcriptional, or post-transcriptional regulation (4–6). LncRNAs can regulate the chromatin structure by directly interacting with RNA, DNA, or proteins. Additionally, lncRNAs modulate several biological processes by functioning as molecular scaffolds or microRNA sponges (7–9). Furthermore, lncRNAs directly modulate the nuclear structure, promote the functions of intracellular molecules by interacting with them, and regulate the transcription or translation of genes (10).

Several studies have demonstrated that some lncRNAs are correlated with the pathogenesis of human diseases, especially cancer (11–13). LncRNAs play a critical role in the oncogenesis and progression of LUAD. UPLA1, a novel lncRNA, promotes the malignant behavior of LUAD by activating the Wnt/β-catenin signaling pathway (14). LINC00628, which is upregulated in LUAD, upregulates the expression of *LAMA3* in LUAD by promoting the methylation of its promoter through the recruitment of DNMT1, DNMT3A, and DNMT3B. Consequently, LINC00628 promotes the proliferation, migration, and invasion of tumor cells and confers resistance to vincristine (15). LncRNA NLUCAT1 is markedly upregulated in the LUAD cell line A549 under hypoxic conditions. The expression of lncRNA NLUCAT1 is regulated by the transcription factors NF-κB and NRF2, which promote proliferation and invasion, regulate the oxidative stress response, and confer cisplatin resistance in the LUAD cells (16). Various lncRNAs regulate the malignancy of LUAD. However, the complex molecular regulatory mechanisms of lncRNAs in LUAD have not been completely elucidated.

This study focused on the lncRNA LINC00958. Previous studies have reported that LINC00958 is involved in the malignant behavior of various solid tumors, including bladder cancer, breast cancer, liver cancer, cervical cancer, and tongue squamous cell carcinoma (17–21). This study aimed to examine the prognostic value of LINC00958 in LUAD and its role in the pathogenesis of LUAD.

## Materials and Methods

### Clinical Data

The RNA sequencing (RNA-Seq) data of LUAD were downloaded from The Cancer Genome Atlas (TCGA) (https://portal.gdc.cancer.gov/), which comprised datasets of 535 LUAD samples and 59 non-tumorous samples. The R package was used to examine the expression of LINC00958 in LUAD. The expression of LINC00958 in LUAD and its correlation with the prognosis of patients with LUAD were analyzed using a tissue microarray (TMA) comprising 98 LUAD samples (Outdo Biotech Co., Ltd.; Cat No. HLugA180Su07; Shanghai, China). The clinicopathological data of 98 patients with LUAD are summarized in **Table 1**. Experiments with TMA were approved by the ethics committee of Shanghai Outdo Biotech Co., Ltd (No. YBM-05-02). The research protocol used in this study was approved by the ethics committee of The First Hospital of Lanzhou University (No. LDYYLL2018-12).

**Table 1:** Correlation between LINC00958 expression and clinicopathological characteristics of LUAD patients.

	Variables	LINC00958 expression		Total	χ^2^	*P* value ^*^
		low	high			
Age(year)					1.718	0.190
	≤66	38	36	74		
	<66	16	8	24		
Sex					0.286	0.593
	Female	25	18	43		
	male	29	26	55		
Smoking history					0.010	0.920
	No	45	37	82		
	Yes	9	7	16		
Grade					1.300	0.254
	I/II	39	27	66		
	III/IV	15	17	32		
Differentiation					4.107	0.128
	Well	19	21	40		
	Moderate	22	19	41		
	Poor	13	4	17		
TNM stage					13.889	<0.001
	I/II	44	20	64		
	III/IV	10	24	34		
T stage					3.543	0.060
	T1/T2	42	28	70		
	T3/T4	12	16	28		
N stage					11.179	0.001
	N0	33	12	45		
	N1/N2/N3	21	32	53		
M stage					1.240	0.265
	M0	54	43	97		
	M1	0	1	1		

^*^P values of age, sex, smoking history, grade, differentiation, TNM stage, T stage, N stage, and M stage was calculated by χ^2^ test.

### Cell Culture

The following cell lines were purchased from the Cell Bank of Chinese Academy of Sciences (Shanghai, China): 293T cells, human lung epithelial cells (BEAS-2B), and human LUAD cell lines (A549, NCI-H1975, H1299, and HCC827). All cells were cultured in Roswell Park Memorial Institute-1640 medium (Gibco, NY, USA) supplemented with 10% fetal bovine serum (FBS; Gibco) and 1% penicillin/streptomycin (Invitrogen, CA, USA) in a humidified incubator (Thermo Scientific, CA, USA) at 37°C and 5% CO_2_.

### Lentiviral Transduction

Short hairpin RNA (shRNA) against LINC00958 (sh-LINC00958) based on LINC00958 sequence listed in GenBank (NR_038904.1), full-length sequence of HOXA1 (NM_153620), and pLVX-shRNA2-Pro-DX vector were obtained from Dianxi Bio Co., Ltd (Shanghai, China). An empty plasmid was used as the control (sh-NC). The cells (2 × 10^5^ cells/well) were seeded in a 24-well plate, cultured to 70% confluency, incubated with 8 μg/mL polybrene (HANBIO; Shanghai, China), and transfected with lentiviral vectors for 48 h. The knockdown of LINC00958 was verified by quantitative real-time polymerase chain reaction (qRT-PCR). The sequence of sh-LINC00958 was as follows: 5′-AATTCAAAAAA GCAGATTAAGCTCTCTCTAATTCTCTT GAAATT AGAGAGAGCTTAATCTGCCG-3′.

### qRT-PCR

Total RNA was extracted using TRIzol reagent (Thermo Scientific). qRT-PCR analysis was performed using AceQ Universal SYBR qPCR master mix (Vazyme, Nanjing, China). The expression of genes was normalized to that of *GAPDH* (internal control for mRNA) or *U6* (internal control for lncRNA). The relative expression levels of genes were determined using the 2^−ΔΔCT^ method. The primers used in qRT-PCR analysis are shown in **Table S1**.

### Subcellular Localization of LINC00958

The cytoplasmic and nuclear fractions of LUAD cells were prepared using the Cytoplasmic & Nuclear RNA Purification Kit (Norgen, CA, USA), following the manufacturer’s instructions. The expression levels of LINC00958, *GAPDH*, and *U6* in the cytoplasmic and nuclear fractions were determined using qRT-PCR. *GAPDH* and *U6* served as internal controls for cytoplasmic and nuclear RNAs, respectively.

### Fluorescence *In Situ* Hybridization (FISH)

FISH analysis was performed using RNAscope-Probe-HS-LINC00958 (Cat No. 478601) by Advanced Cell Diagnostics (CA, USA). Briefly, the TMA was dewaxed, dehydrated, pretreated with hydrogen peroxide for 10 min, and digested with protease. Next, the TMA was hybridized with the probe for 2 h at 40°C. Nuclei were stained with 4′,6-diamidino-2-phenylindole (DAPI; Cell Signaling Technology, MA, USA). The LINC00958 signal was analyzed using the TissueFAXS Spectra panoramic tissue multispectral imaging quantitative analysis system (TissueGnostics, Vienna, Austria). The tissues were classified into the following two groups based on the percentage of cells with the LINC00958 signal (LINC00958 signal-positive cells/total cells): low-expression group, < 5% of cells with LINC00958 signal; high-expression group, > 5% of cells with the LINC00958 signal. Next, the localization of LINC00958 in LUAD cells was analyzed using FISH, following the manufacturer’s instructions. The TMA was denatured at 80°C for 10 min and hybridized with the hybridization solution containing the probe at 37°C for approximately 10 h. Next, the samples were counterstained with DAPI solution (Cell Signaling Technology) and sealed with FixoGum Rubber Cement (EMS, Beijing, China) in the dark. The fluorescence images were captured under a fluorescence microscope (Zeiss Germany, Oberkochen, Germany).

### Cell Counting Kit-8 (CCK-8) Assay

LUAD cells were seeded in a 96-well plate at a density of 5 × 10^3^ cells/well and cultured under standard conditions. The cells in each well were incubated with 10 μL of CCK-8 solution (Dojindo, Kumamoto, Japan). The fluorescence signal was observed once every 24 h. The absorbance at 450 nm was measured using a microplate reader (Thermo Fisher Scientific).

### EdU (5-Ethynyl-2′-Deoxyuridine) Assay

EdU assay was performed using an EdU kit (Roche, Mannheim, Germany), following the manufacturer’s instructions. Briefly, the cells seeded in a 24-well plate were incubated with 200 μL of EdU medium (5 μM) for 2 h and fixed with 50 μL of 4% paraformaldehyde in phosphate-buffered saline (PBS, pH = 7.4) for 30 min at room temperature. The cells were then incubated with 50 μL of glycine (2 mg/mL) for 5 min, 200 μL of osmotic agent (PBS containing 0.5% Triton X-100) for 10 min, and 200 μL of IX Apollo staining solution at room temperature in the dark for 30 min, and rinsed 2–3 times with 200 μL of osmotic agent (PBS containing 0.5% Triton X-100; 10 min/step). The nuclei were stained with DAPI for 5 min and the cells were observed under a fluorescence microscope.

### Terminal Deoxynucleotidyl Transferase Biotin-dUTP Nick End Labeling (TUNEL) Assay

Apoptosis was examined using a TUNEL kit (Roche), following the manufacturer’s instructions. The cells were observed under a fluorescence microscope (Olympus, Tokyo, Japan). The cells with green nuclei were defined as apoptotic cells. The nucleus was stained blue. Apoptotic cells were assessed in eight randomly selected fields at 200× magnification.

### Wound-Healing Assay

The transfected LUAD cells were cultured in complete growth medium until 90% confluency. A 3-mm wound was introduced across the diameter of each plate. Cell migration to the wound area was determined by imaging the cells under an optical microscope (BD Biosciences, CA, USA) after 24 h in six random fields.

### Transwell Assays

The migration and invasion of cells were examined using a 24-well transwell chamber (Corning Costar, NY, USA). The upper chambers were precoated with or without 0.1 mL (300 μg/mL) Matrigel matrix (Corning) for invasion or migration assays, respectively. The LUAD cells were seeded in the upper chambers with serum-free Dulbecco’s modified Eagle medium (DMEM). The bottom chambers were filled with DMEM containing 10% FBS. The cells were incubated for 24 h. The non-migrating cells were gently removed with a cotton swab, whereas the migrating cells were fixed in 4% paraformaldehyde for 15 min, stained with 0.4% crystal violet for 10 min, and imaged under an inverted light microscope (Olympus). The number of migrating cells in five randomly selected visual fields at 100× magnification was counted.

### Animals

Animal experiments were approved by the Animal Care and Use Committee of the First Hospital of Lanzhou University. BALB/c nude mice (aged 4 weeks; average bodyweight 70–80 g) were purchased from SLAC Laboratory Animal Co. Ltd (SLAC, Shanghai, China). All animals were housed individually in a temperature-controlled room under the following conditions: temperature, 22 ± 2°C; humidity, 50%–60%; circadian cycle, 12 h light/dark cycle. LUAD cells transfected with sh-LINC00958 or sh-NC were subcutaneously injected into nude mice (6 mice per group; 5.0 × 10^6^ cells per mouse). The tumor volume was calculated as follows: V = 0.5 × length × width^2^. Tumor weight and volume were monitored on days 14–22 post-transplantation.

### Hematoxylin-Eosin (HE) Staining

The subcutaneous tumor tissues were fixed with formaldehyde, dehydrated using a gradient alcohol series (70%, 80%, 90%, 95%, and 100%; 5 min/step), and cleared twice with xylene for 10 min. The tissues were immersed in wax and embedded in paraffin. The paraffin-embedded tissues were sliced into 4-μm thick sections and placed on glass slides. The samples were baked at 60°C for 1 h and stained with HE solution for 3 min. Next, the sections were dehydrated, cleared, and sealed with neutral gum. Finally, the sections were observed under an optical microscope (Olympus).

### Immunohistochemical (IHC) Analysis

The paraffin-embedded lung cancer tissue sections were dewaxed and hydrated using a gradient ethanol series. Antigen retrieval was performed using a microwave. Endogenous peroxidase activity was inhibited with 3% hydrogen peroxide. The sections were incubated with a rabbit anti-Ki67 antibody (Abcam, ab15580; 1:200; Cambridge, UK) overnight at 4°C, followed by incubation with horseradish peroxidase-conjugated goat anti-rabbit IgG antibody (Abcam, ab6721; 1:10000). Immunoreactive signals were developed with 3,3′-diaminobenzidine. The sections were counterstained with hematoxylin and sealed.

### RNA-Seq

Total RNA was extracted from LUAD cells transfected with sh-LINC00958 and sh-NC using TRIzol reagent (Thermo Scientific), following the manufacturer’s instructions. The RNA-Seq library was prepared using the VAHTS mRNA-seq V2 library prep kit for Illumina^®^ (NR601; Vazyme). RNA-Seq analysis was performed on an Illumina Novaseq 6000 platform.

### RNA Pull-Down Assay

The RNA pull-down assay was performed using the Pierce™ magnetic RNA-protein pull-down kit (Thermo Scientific). Full-length LINC00958 was obtained using *in vitro* transcription with the T7 high yield RNA transcription kit (TR101; Vazyme). Next, the RNA was biotinylated using the Pierce™ RNA 3′ end desthiobiotinylation kit (Thermo Scientific). Cell extracts were incubated with RNAs for 20 min, followed by incubation with nucleic acid-compatible streptavidin magnetic beads (Thermo Scientific). The samples were washed five times and the LINC00958-associated proteins retrieved from the beads were subjected to sodium dodecyl sulfate (SDS)-polyacrylamide gel electrophoresis and silver staining. The proteins collected from the RNA pull-down were quantified using liquid chromatography-tandem mass spectrometry (LC-MS/MS).

### Chromatin Isolation by RNA Purification (ChIRP)

ChIRP was performed using the EZChIP™ kit (EMD Millipore, MA, USA) following standard protocols. The LINC00958-transduced A549 cells were fixed with 1% formaldehyde (Sigma-Aldrich, MO, USA) for 10 min and quenched in 125 mmol/L glycine (Beyotime, Shanghai, China) for 5 min. The cells were washed twice with PBS and resuspended in sonication buffer [20 mmol/L Tris-HCl (pH = 8), 2 mmol/L ethylenediaminetetraacetic acid (EDTA), 1% Triton X-100, 150 mmol/L NaCl, and 1% SDS] (Thermo Scientific). The samples were sonicated using the Bioruptor^®^ PicoSonication system (Diagenode, Seraing, Belgium) for 10 cycles (30 s on/30 s off). Next, the samples were centrifuged for 10 min at 15 000 g and 4°C. The supernatant was incubated with the biotinylated antisense DNA against Gm18840 (Thermo Scientific) at 4°C overnight. The probes used in the ChIRP assay are listed in **Table S2**. The antisense probes of each DNA were used as negative controls. Streptavidin magnetic beads were washed and added to the reaction mixture for 4 h at 4°C. The beads were washed five times with wash buffer (20 mmol/L Tris-HCl (pH 8), 2 mmol/L EDTA, 1% Triton X-100, 300 mmol/L NaCl, and 0.2% SDS) (Thermo Scientific). The samples subjected to ChIRP were eluted using biotin elution buffer (Thermo Scientific) and de-crosslinked with proteinase K (Beyotime) at 65°C overnight. DNA obtained from the ChIRP assay was purified using the QIAquick PCR purification kit (Qiagen GmbH, Hilden, Germany).

### Chromatin-Immunoprecipitation (ChIP)

ChIP analyses were performed using the EZChIP™ kit (EMD Millipore). For each ChIP assay, 1 × 10^6^ cells were fixed in 1% formaldehyde (Sigma-Aldrich) for 10 min at 37°C and washed twice with ice-cold PBS (Beyotime) containing protease inhibitor cocktail (Sigma-Aldrich). The cells were scraped into conical tubes and lysed with SDS lysis buffer (Beyotime) supplemented with a protease inhibitor cocktail. The chromatin-DNA complex was sonicated and sheared to a length of 200–1000 bp. The samples were centrifuged and the supernatant was transferred to a new tube and diluted with ChIP dilution buffer (Beyotime) containing a protease inhibitor cocktail. Protein A agarose beads (Beyotime) were removed from the diluted cell supernatant and agitated for 1 h at 4°C to reduce non-specific background signals. The supernatant fraction was incubated with the anti-MYC (Santa Cruz Biotechnology, Shanghai, China) or anti-MAX (Cell Signaling Technology) antibodies overnight at 4°C with rotation. Mouse IgG served as a negative control, and anti-RNA pol II antibodies (EMD Millipore) were used as the positive control. The reaction mixture was incubated with protein A agarose beads for 1 h at 4°C with rotation to obtain the antibody/histone complexes. The supernatant containing unbound and non-specific DNA was removed by gentle centrifugation. The protein A agarose/antibodies/histone complex was washed on a rotating platform with a low salt immune complex wash buffer (Beyotime), high salt immune complex wash buffer (Beyotime), LiCl immune complex wash buffer (Beyotime), and TE buffer (Beyotime). Elution buffer was used to separate the complexes from the antibodies. DNA samples were recovered through phenol/chloroform (Beyotime) extraction and ethanol precipitation (Beyotime). The DNA samples were PCR-amplified and resolved using agarose gel electrophoresis with a 2% gel (Beyotime). The ChIP-qPCR primer sequences are listed in **Table S3**.

### Luciferase Reporter Assay

The 3′-untranslated region of LINC00958 was amplified using PCR and cloned into the pGL3 luciferase vector (Promega Corporation, WI, USA). A549 cells were co-transfected with firefly luciferase vector (100 ng) and Renilla luciferase expression vector (10 ng) (Promega Corporation) using Lipofectamine 2000, following the manufacturer’s instructions. At 48 h post-transfection, the luciferase reporter assay was performed using the dual-luciferase reporter assay system (Promega Corporation). Renilla luciferase (pRL-TK) served as an internal control for normalization.

### Bioinformatics Analysis

The RNA-Seq data of LUAD obtained from TCGA were analyzed to identify the differentially expressed genes (DEGs). Gene ontology (GO) and Kyoto Encyclopedia of Genes and Genomes pathway enrichment analyses were performed using Enrichr (http://amp.pharm.mssm.edu/Enrichr) (22). The aligned reads were counted using HTseq_count (23). DEGs were identified using DEseq2 (24). GO analysis was performed using the Database for Annotation, Visualization, and Integrated Discovery database (25). Motif enrichment analysis was performed using the Multiple Em for Motif Elicitation suite (26). Gene set enrichment analysis (GSEA) was performed to screen the significantly altered pathways (27). The following datasets were downloaded from the Gene Expression Omnibus (GEO) database: GSM894103, GSM3073948, and GSM3073949. These datasets were used to analyze the transcription factors regulating LINC00958 expression.

### Statistical Analysis

All statistical analyses were performed using GraphPad Prism v8.0. The data are presented as mean ± standard deviation. The means between two groups were compared using the Student’s *t*-test, whereas those between more than two groups were compared using one-way ANOVA. Differences were considered significant at *P* < 0.05 or 0.001.

## Results

### LINC00958 Expression Is Upregulated in LUAD and Associated With Poor Prognosis

**Figure 1:**
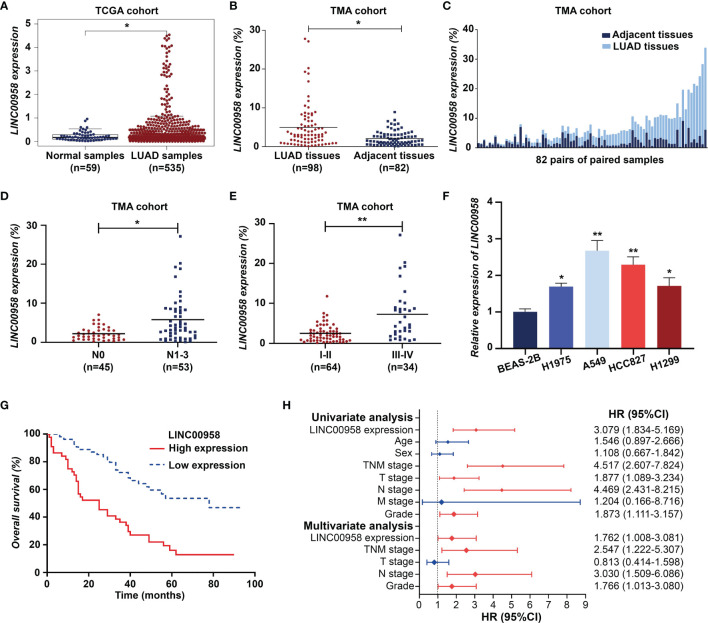
LINC00958 expression is upregulated in patients with lung adenocarcinoma (LUAD) and correlated with poor prognosis. **(A)** The expression of LINC00958 in the LUAD cohort obtained from The Cancer Genome Atlas database. **(B)** Fluorescent *in situ* hybridization (FISH) analysis of tissue microarray demonstrated that the expression of LINC00958 in LUAD tissues was upregulated when compared with that in the adjacent non-tumorous tissues. **(C)** FISH analysis of LINC00958 expression in 82 pairs of LUAD and adjacent non-tumorous tissues. **(D)** The correlation between LINC00958 expression and lymph node metastasis in patients with LUAD. **(E)** The correlation between LINC00958 expression and TNM staging in patients with LUAD. **(F)** The expression level of LINC00958 in LUAD cell lines. **(G)** Kaplan-Meier survival analysis according to the LINC00958 expression levels in 98 patients with LUAD. **(H)** Univariate Cox regression and multivariate analyses revealed that LINC00958 is an independent prognostic factor for LUAD. *P < 0.05 and **P < 0.001.

The RNA-Seq data analysis of the LUAD cohort from TCGA database revealed that the expression level of LINC00958 in LUAD samples was higher than that in non-tumorous samples (**Figure 1A**). As shown in **Figures 1B, C**, FISH analysis revealed that the expression level of LINC00958 in LUAD samples was significantly higher than that in adjacent non-tumorous samples. Additionally, the expression level of LINC00958 was upregulated in the tissues of patients with advanced stage LUAD and those exhibiting lymph node metastasis (**Figures 1D, E**). qRT-PCR analysis revealed that compared with those in the BEAS-2B cells, the expression levels of LINC00958 were significantly upregulated in the LUAD cell lines (**Figure 1F**). In particular, the expression of LINC00958 was the highest in A549 and HCC827 cells. Therefore, these two cell lines were used for further analysis.

Based on the previously established standards, 98 patients with LUAD were classified into low-expression (n = 54) and high-expression groups (n = 44). Next, the correlation between the expression of LINC00958 and the clinical characteristics and prognosis of 98 patients with LUAD was analyzed. As shown in **Table 1**, the upregulated expression of LINC00958 was significantly correlated with TNM staging (*P* < 0.001) and lymph node metastasis (*P* < 0.001) but not with other factors, including age, smoking history, T stage, M stage, and tumor differentiation. Survival analysis of 98 patients with LUAD revealed that the high-expression group was associated with poor prognosis (**Figure 1G**). Univariate Cox regression analysis revealed that the increased risk of death in patients with LUAD was correlated with the upregulated expression of LINC00958, lymphatic metastasis, and TNM staging (**Figure 1H**). Multivariate analysis revealed that the upregulated expression of LINC00958 was an independent prognostic factor for patients with LUAD [Hazards ratio (HR) = 1.762, *P* = 0.047; **Figure 1H**]. These results indicate that LINC00958 expression is upregulated in LUAD and is correlated with poor prognosis.

### *In Vitro* and *In Vivo* Experiments Demonstrated the Oncogenic Role of LINC00958 in LUAD

**Figure 2:**
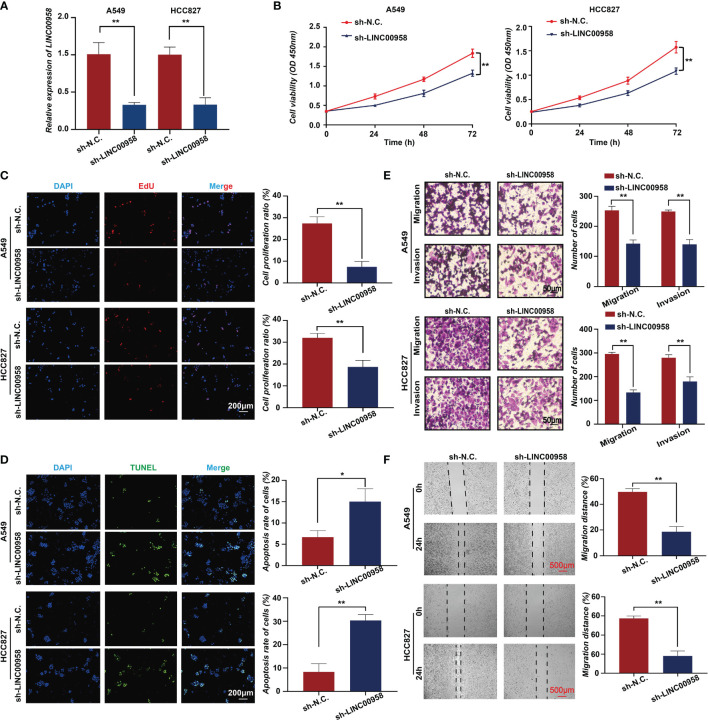
*In vitro* functions of LINC00958 in lung adenocarcinoma (LUAD). **(A)** The silencing efficacy of short hairpin RNA (shRNA) targeting LINC00958 in A549 and HCC827 cells. **(B)** Tumor cell growth was examined using the cell counting kit-8 assays at 0, 24, 48, and 72 h. **(C)** The effect of LINC00958 knockdown on cell proliferation was examined using the 5-ethynyl-2′-deoxyuridine assay. **(D)** Terminal deoxynucleotidyl transferase biotin-dUTP nick end labeling assay was performed to examine the effect of LINC00958 knockdown on cell apoptosis. **(E)** Transwell assay was performed to examine the effect of LINC00958 knockdown on the migration of LUAD cells. **(F)** Wound-healing assay was performed to examine the migration rate of LINC00958 knockdown and control LUAD cells. All assays were repeated three times. * *P* < 0.05, ** *P* < 0.001.

To verify the role of LINC00958 in LUAD, the *in vitro* and *in vivo* functions of LINC00958 were examined. At the cellular level, various bioassays were performed to examine the effect of LINC00958 knockdown on A549 and HCC827 cells. The knockdown efficiency of sh-LINC00958 was validated using qRT-PCR analysis, which revealed that sh-LINC00958 downregulated the expression of LINC00958 by more than 50% (**Figure 2A**). The proliferation of A549 and HCC827 cells transfected with sh-LINC00958 or sh-NC was examined using the CCK-8 assay. LINC00958 knockdown inhibited the growth of A549 and HCC827 cells (**Figure 2B**). The effect of sh-LINC00958 transfection on proliferation and apoptosis was examined using the EdU and TUNEL assays, respectively. As shown in **Figures 2C, D**, the sh-LINC00958-transfected A549 and HCC827 cells exhibited decreased EdU staining signal intensity and increased TUNEL staining signal intensity, which suggested that LINC00958 promotes cell proliferation and inhibits cell apoptosis. Furthermore, the invasion and migration of LINC00958 knockdown A549 and HCC827 cells were examined. As shown in **Figure 2E**, the results of the transwell assay demonstrated that LINC00958 knockdown decreased the invasion and migration of A549 and HCC827 cells. The results of the wound-healing assay (**Figure 2F**) were consistent with those of the transwell assay. The migration rate of LINC00958 knockdown LUAD cells was significantly lower than that of the control cells.

**Figure 3:**
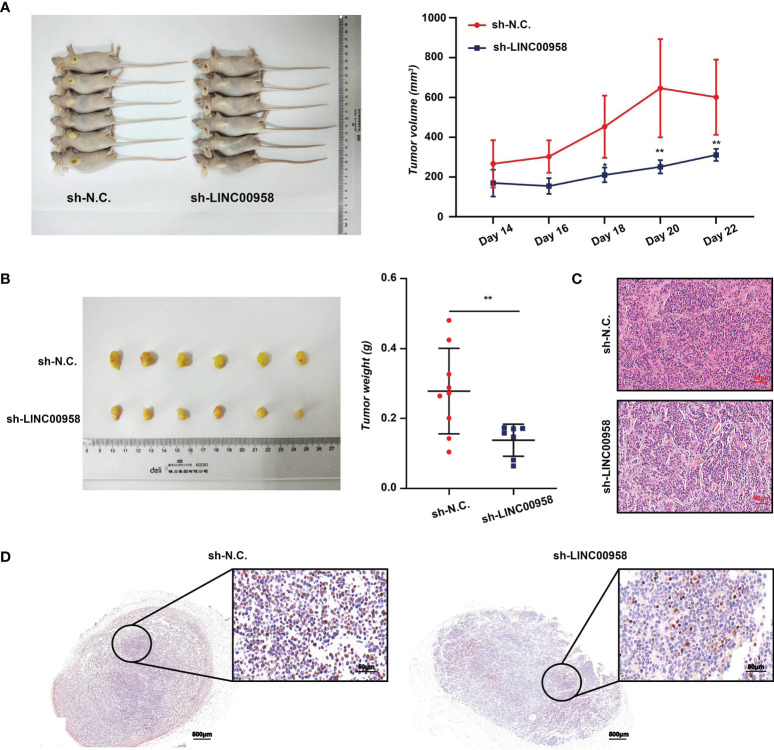
Analysis of *in vivo* functions of LINC00958 in lung adenocarcinoma (LUAD) using a mouse xenograft model. **(A)** LINC00958 knockdown suppressed the tumor growth in xenograft mouse model generated by subcutaneously transplanting A549 cells transfected with sh-LINC00958 or sh-NC. into nude mice. The tumor volume was evaluated on days 14–22 post-transplantation. **(B)** LINC00958 knockdown decreased the tumor size and tumor weight *in vivo*. **(C)** Hematoxylin and eosin (HE)-stained sections of LUAD tumors derived from sh-LINC00958-transfected or sh-NC-transfected cells. **(D)** Representative images of Ki67 immunohistochemical analysis in the tumors derived from sh-LINC00958-transfected or sh-NC-transfected cells. *P < 0.05, **P < 0.001.

A xenograft mouse model was established to examine the effect of LINC00958 knockdown on the cellular phenotype and functions of A549 and HCC827 cells *in vivo.* A549 cells were chosen for the *in vivo* experiments as the phenotype of sh-LINC00958-transfected cells was more pronounced than that of sh-LINC00958-transfected HCC827 cells. Tumor volumes were evaluated from days 14-22 post-transplantation. As shown in **Figures 3A, B**, LINC00958 knockdown decreased tumor volume and size, as well as the tumor weight, in the A549 cell xenograft mouse model. Representative HE-stained tumor tissues of the control and LINC00958 knockdown groups are shown in **Figure 3C**. IHC analysis revealed that the expression of Ki67 in the tumors derived from sh-LINC00958-transfected cells was significantly lower than that in the tumors derived from sh-NC-transfected cells (**Figure 3D**). Thus, the results of *in vivo* experiments were consistent with those of *in vitro* experiments. These findings indicate that LINC00958 has an oncogenic role in LUAD.

### LINC00958 Regulated Metabolism-Related and Immune Response-Related Genes

**Figure 4:**
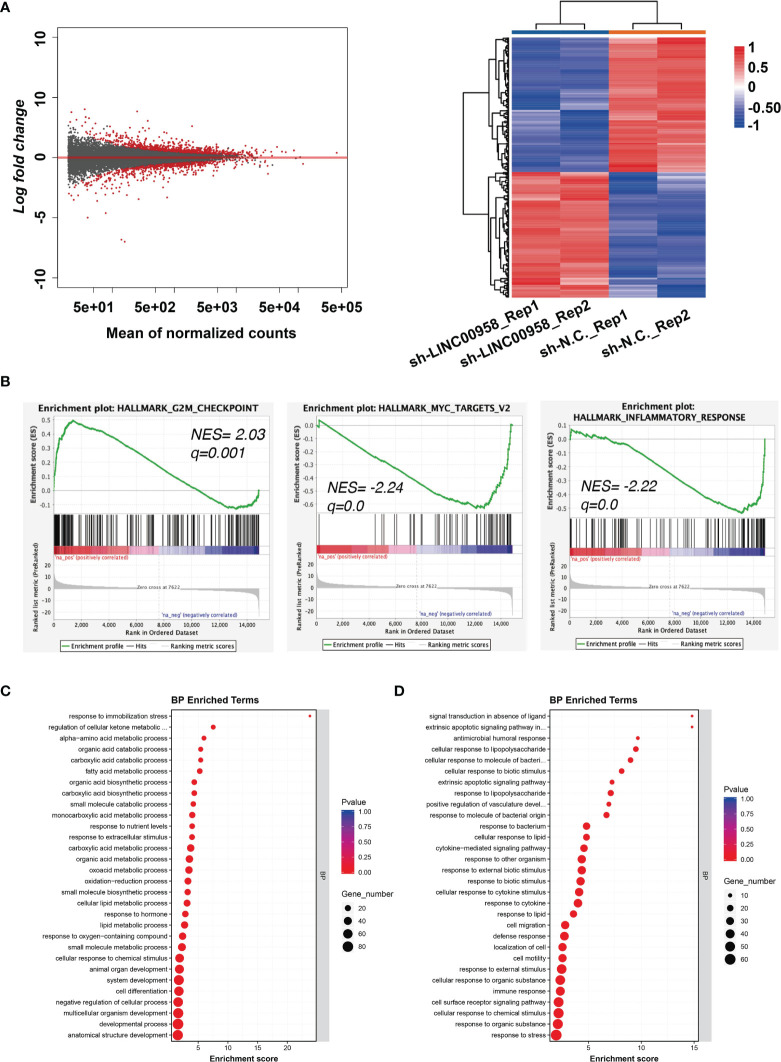
LINC00958 regulated metabolism-related and immune response-related genes. **(A)** Differentially expressed genes in LINC00958 knockdown cells are shown using a volcano plot and heatmap. **(B)** Gene set enrichment analysis of the hallmark gene set in the transcriptome of LINC00958 knockdown and control A549 cells. The enrichment of G2M checkpoint, MYC targets, and inflammatory response was plotted. **(C, D)** Gene Ontology analysis of LINC00958-induced upregulated **(C)** or downregulated **(D)** genes.

RNA-Seq was performed to elucidate the molecular mechanisms of LINC00958 in the pathogenesis of LUAD. **Figure 4A** shows the volcano plots and heatmaps of DEGs between LINC00958 knockdown and control A549 cells. In total, 697 and 612 genes were upregulated, and downregulated, respectively. GSEA of the hallmark gene sets in the transcriptome of LINC00958 knockdown and control A549 cells was performed. The genes were enriched in the G2M checkpoint, MYC targets, and inflammatory responses (**Figure 4B**). The top 30 significantly enriched GO biological processes (BPs), which included fatty acid metabolic processes and alpha-amino acid metabolic processes, of upregulated genes are illustrated in **Figure 4C**. Meanwhile, the top 30 significantly enriched GO BPs, including the process of antimicrobial humoral response and response to cytokines, of downregulated genes are shown in **Figure 4D**, which indicated that the downregulated genes were related to immune response. Therefore, LINC00958 affects the malignant behavior of LUAD cells through different pathways.

### Nuclear Translocation of LINC00958 Indicated a Transcriptional Regulation Role in LUAD Cells

**Figure 5:**
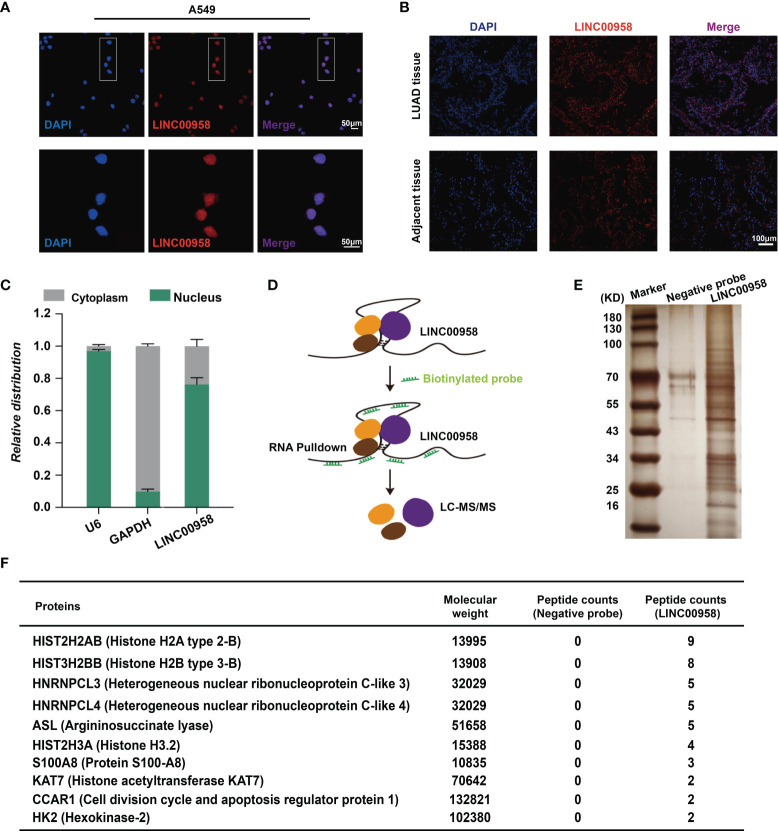
LINC00958 interacted with histones in lung adenocarcinoma (LUAD) cells. **(A)** Distribution of LINC00958 in A549 cells (Scale bar, 50 μm). A549 cells were subjected to fluorescent *in situ* hybridization (FISH) analysis using probes against LINC00958. **(B)** Distribution of LINC00958 in LUAD tissue was analyzed using immunofluorescence (Scale bar, 100 μm). LUAD tissues were subjected to FISH analysis. **(C)** The expression of LINC00958 in A549 cells was examined using quantitative real-time polymerase chain reaction. The expression of LINC00958 in the nuclear and cytoplasmic fractions was determined. *GAPDH* and *U6* were used as markers for the cytoplasmic and nuclear RNAs, respectively. **(D)** Schematic illustration of the RNA pull-down assay. **(E, F)** Silver staining **(E)** and illustration **(F)** of LINC00958-interacting proteins.

To determine the mechanism underlying LINC00958-mediated growth and metastasis of LUAD cells, the A549 cells and LUAD tissues were subjected to FISH analysis using probes against LINC00958 to determine the distribution of LINC00958 in the cytoplasm and nucleus. As shown in **Figure 5A**, the LINC00958 signal (red) was mainly localized to the nucleus (stained blue). Consistently, the LINC00958 signal was localized to the nucleus in the LUAD tissues (**Figure 5B**). The expression levels of LINC00958 in the nuclear and cytoplasmic fractions were further confirmed using qRT-PCR (**Figure 5C**). These results indicated that LINC00958 is involved in transcriptional regulation. Next, an RNA pull-down assay coupled with LC-MS/MS analysis was performed to determine the interactome of LINC00958 in A549 cells (**Figures 5D, E**). LC-MS/MS analysis revealed that LINC00958 directly interacted with 10 proteins (**Figure 5F**). These target proteins were core histone proteins, which further suggested the potential transcriptional regulatory function of LINC00958 in LUAD.

### LINC00958 Reprograms the Transcription of Oncogenes Through HOXA1

**Figure 6:**
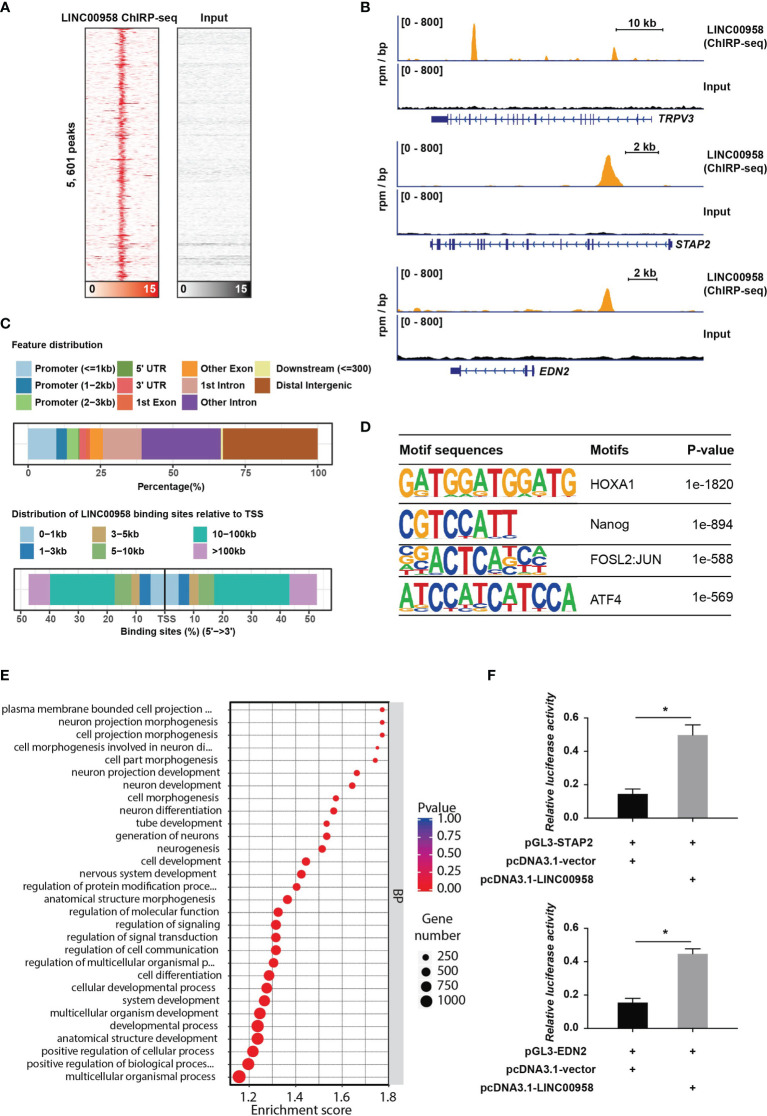
LINC00958 is directly involved in transcriptional regulation. **(A)** Heatmap showing the binding sites of LINC00958 in the chromatin isolation by RNA purification assay. The signals near the peak center of the specific LINC00958-bound regions were plotted. **(B)** Representative illustration of the binding sites of LINC00958 on chromatin. **(C)** Distribution of LINC00958-bound regions on chromatin. The upper panel shows the features of LINC00958-bound peaks. The lower panel shows the distribution of LINC00958-binding sites relative to the transcription start site. **(D)** Motif analysis of LINC00958-bound regions was performed using the Homer2 suite. **(E)** Gene Ontology analysis of LINC00958-bound regions. **(F)** LINC00958 directly regulated the enhancer activity in A549 cells. A549 cells were subjected to a luciferase reporter assay. The LINC00958-bound regions were cloned into the pGL3-promoter vector. The luciferase constructs were co-transfected with the LINC00958-expressing plasmid or empty vector. *P < 0.001.

To explore the direct targets of LINC00958-mediated transcription regulation, ChIRP-Seq was performed. The ChIRP-Seq signal of the binding sites of LINC00958 is shown in **Figure 6A**. The signals near the peak center of each LINC00958-bound region are plotted. In total, 5601 targets of LINC00958 that directly bind to chromatin regions were identified. As shown in **Figure 6B**, LINC00958 directly binds to various chromatin regions, such as TRPV3, STAP2, and EDN2. Of these 5601 LINC00958 targets, 29% and 44.8% were promoters and genes (including exons and introns), respectively (**Figure 6C**). Next, motif enrichment analysis of LINC00958-bound regions was performed using the Homer2 suite. The motifs of HOXA1, NANOG, FOSL2, JUN, and ATF4, were significantly enriched, which indicated that LINC00958 may influence the transcriptional activity of these factors by regulating gene expression (**Figure 6D**). Furthermore, GO analysis revealed that LINC00958-bound regions were enriched in various GO BPs, including cell development, morphogenesis, and signal regulation (**Figure 6E**). The results of the luciferase reporter assay revealed that LINC00958 directly regulated enhancer activity in A549 cells (**Figure 6F**). These results indicate that LINC00958 is involved in transcriptional regulation by directly binding to regulatory regions.

**Figure 7:**
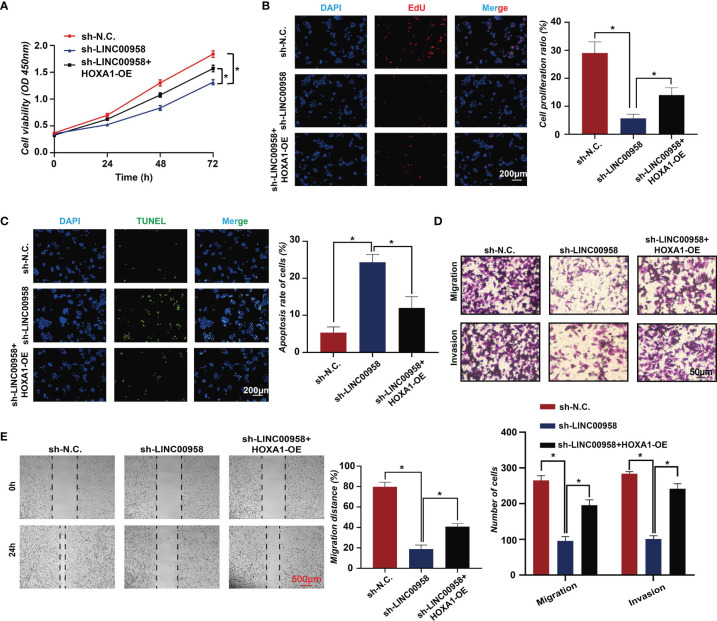
HOXA1 overexpression partially mitigates the oncogenic function of LINC00958. **(A)** Cell counting kit-8 assay results of LINC00958 knockdown, HOXA1-overexpressing LINC00958 knockdown, and control A549 cells at 0, 24, 48, and 72 h. **(B)** The proliferation of LINC00958 knockdown, HOXA1-overexpressing LINC00958 knockdown, and control A549 cells was examined using the 5-ethynyl-2′-deoxyuridine incorporation assay. **(C)** Terminal deoxynucleotidyl transferase biotin-dUTP nick end labeling assay was performed to examine the apoptosis rate of LINC00958 knockdown, HOXA1-overexpressing LINC00958 knockdown, and control A549 cells. **(D)** Transwell assay was performed to examine the invasion and migration of LINC00958 knockdown, HOXA1-overexpressing LINC00958 knockdown, and control A549 cells. **(E)** Wound-healing assay was performed to examine the migration rate of LINC00958 knockdown, HOXA1-overexpressing LINC00958 knockdown, and control A549 cells. All assays were repeated three times. *P < 0.001.

To further validate the LINC00958-mediated reprogramming of transcriptional regulation, rescue assays were performed. HOXA1 is an oncogenic gene play an important role in cancers, including esophageal cancer (28), breast cancer (29, 30), prostate cancer (31), etc. However, there is few research about HOXA1 in LUAD. As seen in **Figure 6D**, the motifs of HOXA1 were most significantly enriched (*P* =1e-1820). Hence, we selected HOXA1 as a specific gene for further experiments by overexpressing HOXA1 in LUAD cells. The CCK-8 assay results revealed that HOXA1 overexpression partially mitigated the LINC00958 knockdown-induced impaired A549 cell growth (**Figure 7A**). Consistent with the cell proliferation phenotype, the results of the EdU (**Figure 7B**) and TUNEL (**Figure 7C**) assays revealed that HOXA1 overexpression mitigated the LINC00958 knockdown-induced decreased proliferation and enhanced apoptosis. The migration of HOXA1-overexpressing LINC00958 knockdown cells was higher than that of LINC00958 knockdown cells (**Figure 7D**). Similarly, the results of the wound-healing assay (**Figure 7E**) revealed the migration rate of HOXA1-overexpressing LINC00958 knockdown cells was higher than that of LINC00958 knockdown cells, which indicated that HOXA1 plays a pivotal role in LINC00958-mediated lung carcinogenesis. These results indicate that LINC00958 is involved in oncogenic transcriptional regulation through HOXA1.

### MYC and MAX Upregulate LINC00958

**Figure 8:**
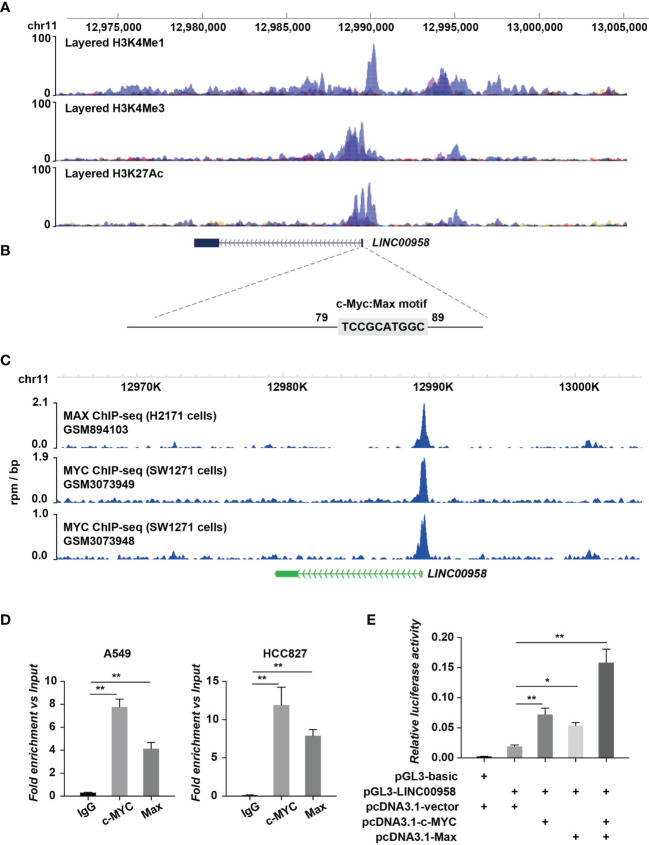
MYC/MAX upregulated LINC00958 expression. **(A)** Transcriptional regulatory regions of LINC00958. The diagram indicated the binding signals of the layered H3K27ac, H3K4me1, and H3K4me3 in the internal transcriptional unit or the proximal region of LINC00958. The layered histone markers were obtained from the ENCODE Regulation database. **(B)** Motif analysis revealed an MYC/MAX motif at the regulatory region of LINC00958. The sequences of regions with high H3K4me1, H3K4me3, and H3K27ac signal intensities were extracted for motif analysis. **(C)** MYC/MAX was bound to the promoter regions of LINC00958. Chromatin immunoprecipitation (ChIP) sequencing data that identified MYC and MAX at the regulatory regions of LINC00958 in lung cancer cells, such as H2171 and SW1271 cells, were retrieved from the Gene Expression Omnibus database (GSM894103, GSM3073948, and GSM3073949) and plotted. **(D)** ChIP-quantitative polymerase chain reaction analysis (ChIP-qPCR) revealed that MYC/MAX was bound to the promoter region of LINC00958 in A549 and HCC827 cells. A549 and HCC827 cells were subjected to ChIP-qPCR using anti-MYC and anti-MAX antibodies. IgG was used as the negative control. Relative enrichment versus 2% input was plotted. **(E)** MYC and MAX trans-activated the promoter activity of LINC00958. Relative firefly luciferase activity versus Renilla luciferase activity was plotted in columns. *P < 0.01, **P < 0.001.

Next, the regulation of LINC00958 by transcription factors was examined using the ChIP-Seq assay. The transcriptional regulatory regions of LINC00958 are shown in **Figure 8A** in which the binding signals of the layered H3K27ac, H3K4me1, and H3K4me3 within or near LINC00958 are presented. Moreover, motif enrichment analysis revealed the MYC/MAX motif in the regulatory region of LINC00958 (**Figure 8B**). The ChIP-Seq data of MYC and MAX at the regulatory regions of LINC00958 in lung cancer cells, including H2171 and SW1271 cells, were retrieved from the GEO database (GSM894103, GSM3073948, and GSM3073949) and plotted (**Figure 8C**). MYC/MAX was bound to the promoter region of LINC00958. Next, the A549 and H1299 cells were subjected to ChIP-qPCR using anti-MYC and anti-MAX antibodies to examine if MYC and MAX bind to the promoter region of LINC00958 (**Figure 8D**). The results of the luciferase assay revealed that MYC and MAX trans-activated the promoter of LINC00958 (**Figure 8E**).

## Discussion

LncRNAs, which bind to DNA, RNA, or proteins, are epigenetic factors involved in the progression of tumors, including lung cancer (32). However, the regulatory functions of these lncRNAs have not been elucidated. This study reported the clinical significance, *in vivo* and *in vitro* functions, and mechanisms of LINC00958 in LUAD. Consistent with the previously reported function of LINC00958 as an oncogene in other cancer types (18, 19, 33–40), this study demonstrated that LINC00958 promotes lung cancer growth and metastasis and inhibits cancer cell apoptosis. This suggested that LINC00958 has a potent tumorigenesis role in cancer. The expression of LINC00958 was upregulated in clinical tumor samples. Further validation of LINC00958 expression in a large number of samples and other body fluids, such as blood, may enable the application of LINC00958 as a diagnostic marker for LUAD. Additionally, the high LINC00958 expression was positively correlated with TNM staging and lymph node metastasis, which is consistent with its role in promoting cancer cell migration and invasion. Furthermore, LINC00958 also serves as an independent prognostic factor for patients with LUAD. Thus, LINC00958 has potential clinical applications to predict the prognosis of patients with LUAD.

Several studies have demonstrated that lncRNAs promote lung cancer progression by regulating gene expression at transcriptional and post-transcriptional levels (41), which is consistent with the biological functions of LINC00958 demonstrated in this study. LINC00958 is reported to function as a miRNA sponge and regulate target gene expression. For example, LINC00958 sponges miR-625 and suppresses miR-625-mediated NUAK1 inhibition in nasopharyngeal carcinoma and miR-625-mediated LRRC8E inhibition in cervical cancer (18). Additionally, LINC00958 regulates the miR-203/CDK2 axis in glioma (42), the miR-378a/IGF1R axis in bladder cancer (39), the miR-627/YBX2, and miR-185-5p/YWHAZ axes in oral squamous cell carcinoma (40), miR-5095/RRM2 in cervical cancer (18), miR-106a/AKT/mTOR in head and neck squamous cell carcinoma (36), and the miR-3619/HDGF axis in hepatocellular carcinoma (19). To examine if LINC00958 functions are dependent on competing endogenous RNA, the cellular localization of LINC00958 in LUAD cell lines and tumor tissues was examined. LINC00958 was mainly localized (>70%) to the nucleus with a minor proportion localized (<30%) to the cytoplasm. The RNA pull-down assays identified various LINC00958-bound proteins, which supported the subcellular localization of LINC00958. These findings suggest that LINC00958 regulates transcription in LUAD.

LncRNAs are reported to function as transcriptional regulators in lung cancer by recruiting and interacting with histone modifiers, DNA methyltransferases, and transcription factors (43–48). For example, CASC9 promotes gefitinib resistance in non-small cell lung cancer (NSCLC) by recruiting EZH2 (a histone methyltransferase) and inducing the epigenetic repression of DUSP1 (49). Similarly, lncRNA FOXC2-AS1 promotes NSCLC oncogenesis by repressing p15 expression through interaction with EZH2 (50). Han et al. reported that LINC00857 recruits NF-κB1 to the *BIRC5* promoter region and increases BIRC5-dependent radiosensitivity of LUAD (51). Guo et al. reported that LINC00163 impaired lung cancer development by recruiting ARID1A to the *TCF21* promoter (52). LINC00337 silences *TIMP2* expression by recruiting the epigenetic repressor DNMT1 to its promoter region and promotes NSCLC progression (53). In this study, LINC00958 interacted with proteins involved in transcriptional regulation and was directly bound to the regulatory regions of genes associated with angiogenesis and migration. Motif analysis identified transcription factors, such as HOXA1, NANOG, FOSL2, JUN, and ATF4 in the LINC00958-bound regions, which partially validated the LINC00958-mediated transactivation of p-c-JUN. HOXA1 encodes a DNA-binding transcription factor that regulates gene expression, morphogenesis, and differentiation. Previous studies have reported that HOXA1 contributes to cancer pathogenesis and progression, immunosuppressive activity of myeloid-derived suppressor cells, and therapeutic response (54–60). The results of the rescue assays indicated that LINC00958 promotes lung cancer progression by regulating the transcriptional activity of HOXA1. Similar to that of lncRNAs, such MALAT1, the miRNA sponging function of LINC00958 localized in the cytoplasm may promote lung cancer progression (61–64).

Additionally, ChIP-Seq and luciferase assay results indicated the mechanisms underlying LINC00958 upregulation in LUAD. MYC/MAX was a hub regulator of LINC00958, which is consistent with the results of studies on head and neck squamous cell carcinoma cells (65). Yang et al. reported that the transcription factor SP1 upregulates LINC00958 expression in LUAD (66). As the oncogenic function of LINC00958 has been reported in multiple cancer types, MYC/MAX, a vital oncogenic transcription factor in various cancers (67–69), may contribute to the transcriptional regulation of LINC00958 in cancer. The expression of LINC00958 in hepatocellular carcinoma was also influenced by METTL3 in an N6 methylation-dependent manner (19). Gene expression is modulated at multiple levels in the tissues. Further studies are needed to examine the regulation of LINC00958 by other LUAD-specific modulators.

LINC00958 knockdown affected the expression of genes related to immune responses. Additionally, LINC00958 was bound to the regulatory regions of genes related to cytokine production. Thus, LINC00958 may also be involved in the communication between cancer cells and tumor immune microenvironment. The HOXA1 motif was significantly enriched in the LINC00958-bound regions, which suggested that LINC00958 may regulate the targets of HOXA1. The transcription factor HOXA1, which regulates genes related to cell proliferation, differentiation, and immune response, has been reported to contribute to the pathogenesis of several malignancies (70), including lung cancer (60, 61, 71). Thus, LINC00958 may be involved in multiple processes through HOXA1. However, further studies are needed to elucidate the underlying mechanisms.

This study has some limitations. More than one cell line should be performed to identify or verify the targets. The function of LINC00958 in LUAD was examined based on loss-of-function studies. However, LINC00958 overexpression and rescue assays must be performed to elucidate the LINC00958 functions. This study demonstrated that LINC00958 promoted LUAD progression by transcriptional reprogramming. However, further studies are needed to validate the binding of LINC00958 to transcriptional regulators. Additionally, factors other than MYC/MAX that modulate the expression of LINC00958 and the underlying mechanisms must be examined in the future.

**Figure 9:**
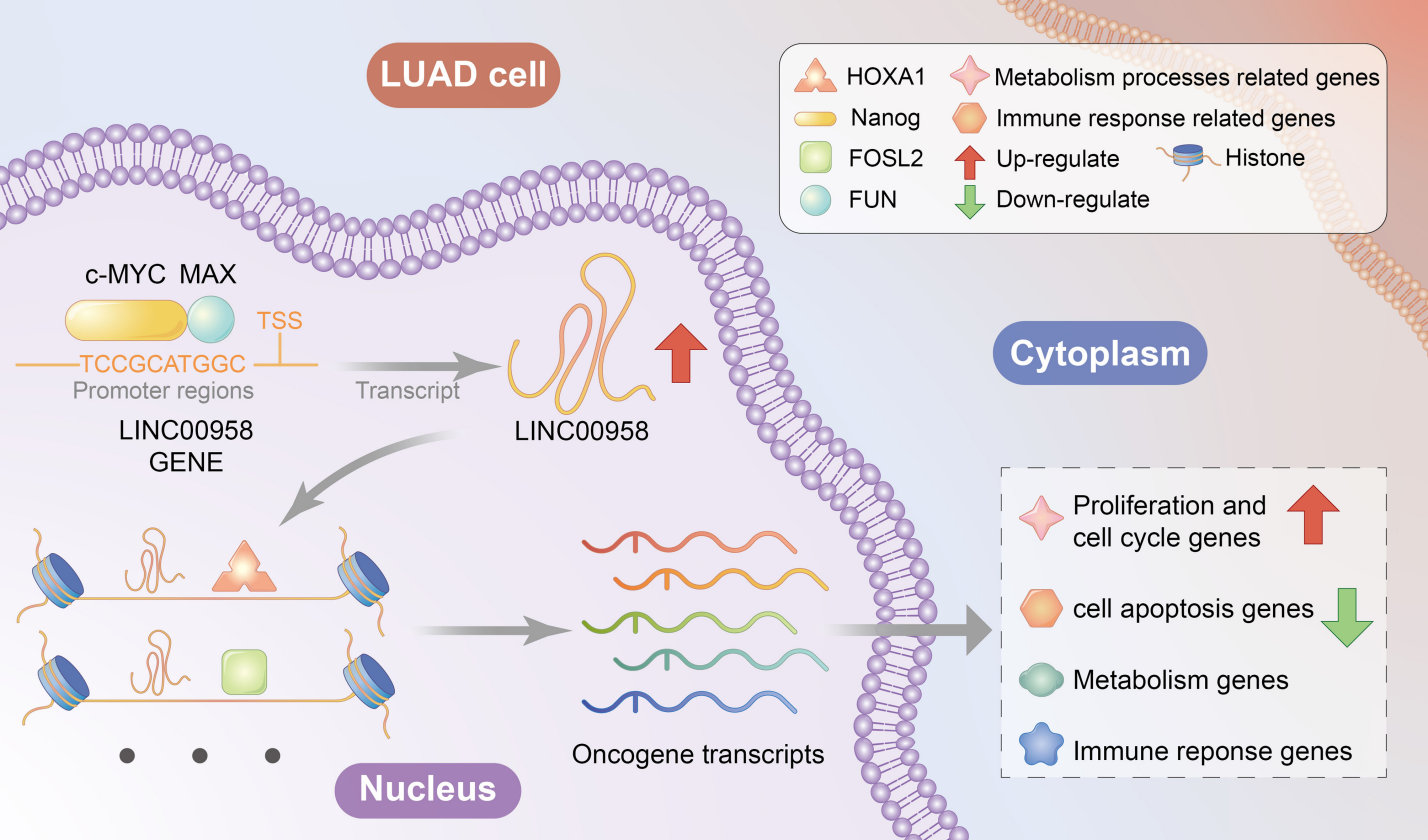
Proposed mechanism of LINC00958.

The findings of this study indicate that MYC/MAX-trans-activated LINC00958 enhances the malignant behavior of LUAD by recruiting the transcriptional regulator HOXA1 and inducing oncogenic reprogramming (**Figure 9**).

## Data Availability Statement

The raw data supporting the conclusions of this article will be made available by the authors, without undue reservation.

## Ethics Statement

The studies involving human participants were reviewed and approved by the ethics committee of The First Hospital of Lanzhou University (No. LDYYLL2018-12). The patients/participants provided their written informed consent to participate in this study. The animal study was reviewed and approved by the ethics committee of The First Hospital of Lanzhou University.

## Author Contributions

TZ, DZ, X-mH, and BZ and designed the study. TZ, FS, Y-bL, and X-lL collated the data, carried out data analyses, and produced the initial draft of the manuscript. TZ, FS, X-lL, H-yD, and T-nY conducted experiments. All authors have read and approved the final submitted manuscript.

## Funding

This work was supported by funding from (1) National Natural Science Foundation of China (81802269); (2) Construction Project of Clinical Medical Research Center from Gansu Provincial Department of Science and Technology (21JR7RA390); (3) Natural Science Foundation of Gansu Province (20JR5RA352, 20JR10RA686 and 21JR7RA386); (4) Gansu Province Higher Education Innovation Ability Improvement Project (2019B-009 and 2020B-009); (5) Scientific and Technological Development Guiding Plan Project of Lanzhou City (2020-ZD-74); (6) Subsidy Project of The First Hospital of Lanzhou University (ldyyyn2018-13, ldyyyn2018-43, ldyyyn2019-18 and ldyyyn2019-19).

## Conflict of Interest

The authors declare that the research was conducted in the absence of any commercial or financial relationships that could be construed as a potential conflict of interest.

## Publisher’s Note

All claims expressed in this article are solely those of the authors and do not necessarily represent those of their affiliated organizations, or those of the publisher, the editors and the reviewers. Any product that may be evaluated in this article, or claim that may be made by its manufacturer, is not guaranteed or endorsed by the publisher.
