# Supplementary material for: MYC/MAX-Activated LINC00958 Promotes Lung Adenocarcinoma by Oncogenic Transcriptional Reprogramming Through HOXA1 Activation
Source: Front Oncol. 2022 Feb 9;12:807507. doi: 10.3389/fonc.2022.807507 (PMC8864111; doi:10.3389/fonc.2022.807507)
Supplement: Supplementary file 1 [file DataSheet_1.pdf]

Table S1. The primers forward and primers reverse of qRT-PCR

Gene name	Primer Forward	Primer Reverse
LINC00958	CTCAGAGGGCTGTTCTCCTG	GCAAGATAGCTCCAGGTTGG
U6	CTCGCTTCGGCAGCACA	AACGCTTCACGAATTTGCGT
GAPDH	GGAGCGAGATCCCTCCAAAAT	GGCTGTTGTCATACTTCTCATGG

Table S2. The probes used in the ChIRP assay

Probes	Sequences
LINC00958-Positive-1	ggaagcaagcagtttcttcaca
LINC00958-Positive-2	ggagagcttaaactttctccct
LINC00958-Positive-3	ctgcaggagtgattgcttctcc
LINC00958-Positive-4	tgatggatgagtggatccctct
LINC00958-Positive-5	atcagtggactcatctttgcct
LINC00958-Positive-6	cttggtagctgagtctctccac
LINC00958-Positive-7	cctttttattgctcctcccgtg
LINC00958-Positive-8	cagagtgacacatgctacttaa
LINC00958-Negative	tgtgaagaaactgcttgcttcc

Table S3. The ChIP-qPCR primer sequences

**Gene**	Sequence of promoter
LINC00958	F: 5’-TGAGAGGGAAGCAAGCAGTT-3’<br>R: 5’-GTGGGAGGTGACTCAATCGT-3’
